# Estimating Adverse Events Associated With Herbal Medicines Using Pharmacovigilance Databases: Systematic Review and Meta-Analysis

**DOI:** 10.2196/63808

**Published:** 2024-08-29

**Authors:** Chuenjid Kongkaew, Dang Thuc Anh Phan, Prathan Janusorn, Pajaree Mongkhon

**Affiliations:** 1 Centre for Safety and Quality in Health Department of Pharmacy Practice Faculty of Pharmaceutical Sciences, Naresuan University Phitsanulok Thailand; 2 Department of Pharmaceutical and Biological Chemistry School of Pharmacy University College London London United Kingdom; 3 Faculty of Pharmacy Hue University of Medicine and Pharmacy Hue City Vietnam; 4 Pharmacy Department Soidao Hospital Chantaburi Thailand; 5 School of Pharmaceutical Sciences University of Phayao Phayao Thailand

**Keywords:** herbal medicine, pharmacovigilance, adverse event, spontaneous reporting system, meta-analysis

## Abstract

**Background:**

Herbal medicines (HMs) are extensively used by consumers/patients worldwide. However, their safety profiles are often poorly reported and characterized. Previous studies have documented adverse events (AEs) associated with HMs, such as hepatotoxicity, renal failure, and allergic reactions. However, the prevalence rate of AEs related to HMs has been reported to be low. To date, no systematic review and meta-analysis has comprehensively analyzed the AEs of HMs using published data acquired from pharmacovigilance (PV) databases.

**Objective:**

This study aimed to (1) estimate the reporting rate of the AEs of HMs using PV databases and (2) assess the detailed data provided in AE reports.

**Methods:**

In this systematic review and meta-analysis, MEDLINE/PubMed, SCOPUS, EMBASE, and CINAHL were systematically searched for relevant studies (until December 2023). The DerSimonian-Laird random effects model was used for pooling the data, and subgroup analyses, the meta-regression model, and sensitivity analysis were used to explore the source of heterogeneity. Crombie’s checklist was used to evaluate the risk of bias (ROB) of the included studies.

**Results:**

In total, 26 studies met the eligibility criteria. The reporting rate of the AEs of HMs ranged considerably, from 0.03% to 29.84%, with a median overall pooled estimate of 1.42% (IQR 1.12%-1.72%). Subgroup analyses combined with the meta-regression model revealed that the reporting rate of the AEs of HMs was associated with the source of the reporter (*P*=.01). None of the included studies provided full details of suspected herbal products, only the main ingredients were disclosed, and other potentially harmful components were not listed.

**Conclusions:**

This systematic review and meta-analysis highlighted risks related to HMs, with a wide range of reporting rates, depending on the source of the reporter. Continuous efforts are necessary to standardize consumer reporting systems in terms of the reporting form, education, and follow-up strategy to improve data quality assurance, aiming to enhance the reliability and utility of PV data for monitoring the safety of HMs. Achieving effective monitoring and reporting of these AEs necessitates collaborative efforts from diverse stakeholders, including patients/consumers, manufacturers, physicians, complementary practitioners, sellers/distributors, and health authorities.

**Trial Registration:**

PROSPERO (Prospective International Register of Systematic Reviews) CRD42021276492

## Introduction

The World Health Organization (WHO) defined herbal medicines (HMs) as substances that “[…]contain active ingredient parts of plants or other plant materials or combinations thereof” [1]. These substances have been used as part of traditional (folk) medicine over the millennia, and they are becoming increasingly popular in recent years [1,2]. More than 80% of the population worldwide relies on traditional herbal treatment, and those estimates differ by country, ethnicity, age group, gender, or clinical condition [3-5]. There are many reasons for this high consumption, including escalating costs of health care, barriers to physician consultations, personal preferences, and perceived safety and health benefits of HMs. Most consumers/patients assume that HMs are of natural origin and believe them to be harmless [6]. Contrary to this fallacy, there have been reports of serious adverse events (AEs) associated with HMs, including hepatotoxicity, renal failure, allergic reactions, colon perforation, carcinoma, coma, and even death [7,8]. These AEs can be attributed to overdosing, adulteration, or contamination of HMs, herb-drug interactions, and herb-herb interactions [9-12].

Pharmacovigilance (PV) is the science and activities relating to the detection, assessment, understanding, and prevention of the adverse effects of drugs or any other possible drug-related problems [2]. Essentially, a PV system aims to avert AEs resulting from medication use and implement measures to minimize the consequences of potential adverse effects [2,13]. Over recent decades, the assessment of medication safety and benefits has been significantly transformed by the development of large databases and statistical programs, enhancing the use and rapid analysis of data [14]. Collecting and analyzing individual reports on AEs remains the most cost-effective and straightforward method for drug safety assessment and new signal detection [13].

Individuals reporting AEs have been used as sources of data on the safety of medicinal products. These reporters may include health care professionals (HCPs), such as physicians, dentists, pharmacists, and nurses, who possess medical qualifications. Additionally, consumers (non-HCPs), such as patients, patients’ relatives, and caregivers, are now recognized as valuable sources of safety information about medicinal products [2,13].

In light of increasing safety concerns, many researchers advocate for the integration of herbal products into the existing PV system and the use of a single reporting form [15]. Recently, the PV of HMs, known as phytovigilance, has garnered attention [6], with spontaneous reporting identified as the primary method for monitoring these products [2,16].

To the best of our knowledge, no similar systematic review and meta-analysis evaluating the reporting rate of the AEs of HMs using the data acquired from PV databases has been published. In addition, little is known about the quality of information provided within the AE reports through a spontaneous reporting system. Therefore, the aim of this systematic review and meta-analysis was to estimate the reporting rate of the AEs of HMs using data from PV databases and to assess the detailed information provided in AE reports. The study also aimed to scrutinize the characteristics of these AEs, including their severity, causality, and outcomes.

## Methods

### Search Strategy

This systematic review and meta-analysis was registered with PROSPERO (International Prospective Register of Systematic Reviews; ID CRD42021276492) and conducted by adhering to the *Cochrane Handbook for Systematic Reviews of Interventions* and the PRISMA (Preferred Reporting Items for Systematic Reviews and Meta-Analyses) guidelines. The MEDLINE/PubMed, EMBASE, CINAHL, and SCOPUS databases were searched from their inception until December 2023 by 2 researchers (authors DTAP and PJ). Additional relevant papers were also obtained through a manual search by checking each included study’s references. The gray literature, such as abstracts, conference proceedings, and editorials, was excluded. The keywords and their synonyms for the search strategy were as follows: (“pharmacovigilance” OR “post-marketing surveillance” OR “adverse event reporting” OR “adverse event reporting system” OR “adverse event reports” OR “self-reporting”) AND (“herbal medicine” OR “herbal remedies” OR “medicinal herb” OR “herbal drugs” OR “herbal products” OR “botanical medicines” OR “phytomedicine” OR “phytotherapy” OR “traditional medicine”). In this study, the term “herbal medicines” was defined as “herbs, herbal materials, herbal preparations and finished herbal products. Herbs include crude plant material (leaves, flowers, fruit, seeds, stems, wood, bark, roots, rhizomes or other plant parts, which may be entire, fragmented or powdered). Herbal materials include, alongside herbs, fresh juices, gums, fixed oils, essential oils, resins and dry powders of herbs. Herbal preparations include comminuted or powdered herbal materials, or extracts, tinctures and fatty oils of herbal materials. Finished herbal products consist of herbal preparations made from one or more herbs” [2].

### Study Selection

Primary studies were included in this research if they were original studies reporting AEs associated with HMs through a voluntary reporting scheme, with no restriction of language. Studies were excluded if they (1) were reviews or systematic reviews, case reports, conference abstracts, or editorials; (2) were studies reporting AEs on animals; (3) did not provide AEs in detail; or (4) were studies reporting AEs associated with complementary and alternative medicines.

The primary outcome was the reporting rate of the AEs of HMs, calculated as the number of reports referred to AEs associated with HMs (numerator) divided by the total number of reports of AEs associated with all medicines (denominator).

Studies that could not be included in the meta-analysis because of incomplete data were reviewed narratively. Endnote 20 (Clarivate Analytics) was used as a reference manager to import citations and remove duplicate publications.

### Data Screening and Extraction

Titles and abstracts were screened independently by 2 researchers (DTAP and PJ). The full text was then examined in detail by the same 2 researchers. Non-English papers were translated using Google Translate [17].

The following data were independently double-extracted into evidence tables by 2 researchers (authors CK and DTAP): general characteristics of the studies (eg, country of study, source of databases, study period) and outcome data (eg, reporting rate of AEs of HMs, severity of AEs, causality assessment, affected body parts or systems most involved in AEs).

### Risk-of-Bias Assessment

Two independent investigators (DTAP and PJ) assessed the risk of bias (ROB) using Crombie’s checklist for cross-sectional studies [18,19]. The following items were appraised: (1) appropriate design, (2) adequate description of data, (3) reported response rates, (4) adequate representation of the total sample, (5) clearly stated aims and the likelihood of reliable and valid measurements, (6) statistical significance, and (7) adequate description of analyses. Each item scored 1 point for yes, 0.5 points for unclear, and 0 for no. The total score was modified as high ROB (0-4), moderate ROB (>4 and <6), and low ROB (6-7). Any discrepancies during data extraction and ROB assessment were resolved by consensus between these investigators and the principal investigator (CK). The κ statistic was used to evaluate the degree of agreement between investigators. A negative κ indicated a lack of agreement, while the following ranges were used to interpret the level of agreement: 0.01-0.20 as none to slight, 0.21-0.40 as fair, 0.41-0.60 as moderate, 0.61-0.80 as substantial, and 0.81-1.00 as almost perfect agreement [20].

### Data Synthesis and Statistical Analysis

Pooled effect estimates for the reporting rate of the AEs of HMs across the included studies with corresponding 95% CIs were calculated as a percentage using the DerSimonian-Laird random effects model [21]. To assess the heterogeneity among studies, standard *χ*^2^ tests and the *I*^2^ statistic were used [22]. Where high heterogeneity was indicated (*I*^2^≥75%), the results across studies were summarized using the median reporting rate and the IQR. To explore the possible sources of heterogeneity, subgroup analyses were performed by continent (North America, Europe, Asia, Africa), the source of the reporter (consumer, HCPs, or all stakeholders), source of databases (poison control center, national PV center, or regional PV center), and the ROB. Sensitivity analysis was performed to examine the influence of studies with a high ROB and the source of the reporter to the pooled estimate of the reporting rate of AEs associated with HMs. A univariate random effects meta-regression was also used to investigate heterogeneity. Egger’s asymmetry test was conducted to look for signs of publication bias [23]. If significant publication bias existed, the trim-and-fill method was performed to adjust the publication bias [23]. Statistical tests were 2-sided with a significance of *P*<.05. All statistical analyses were performed with STATA 17.0 (Stata Corp LLC).

## Results

### Search Outcomes

**Figure 1:**
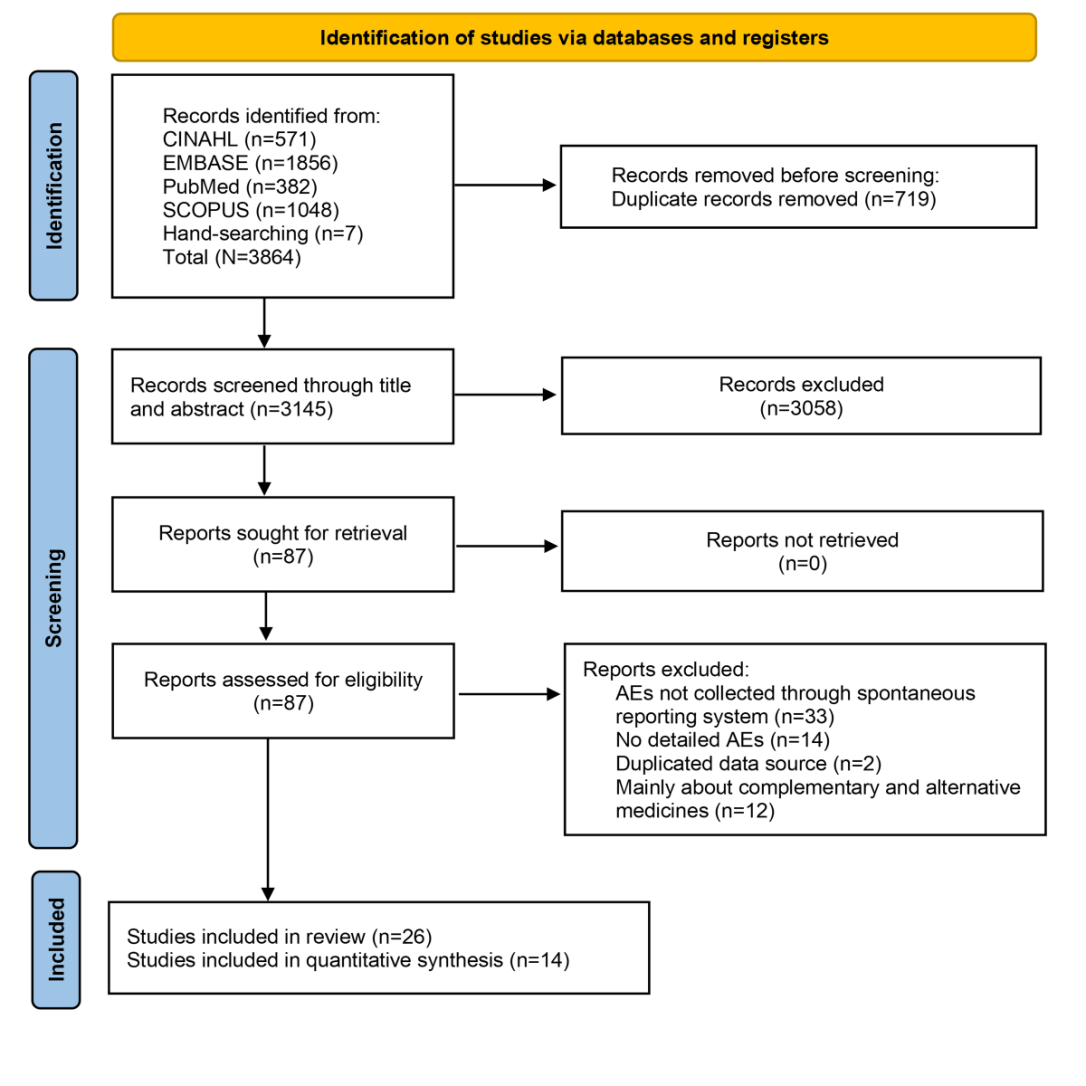
PRISMA flow diagram. AE: adverse event; PRISMA; Preferred Reporting Items for Systematic Reviews and Meta-Analyses.

There were 3864 papers identified from the databases (n=3857, 99.8%, papers) and manual searching (n=7, 0.2%, papers). After removing duplicates, 3145 (81.4%) papers were screened against the eligibility criteria. Screening of titles or abstracts resulted in 3058 (97.2%) papers being excluded. A total of 87 (2.8%) papers remained for full-text screening. Of those, 61 (70.1%) studies were excluded because of AEs not collected through voluntary reporting schemes (n=33, 54.1%, papers), detailed AEs not provided (n=14, 23%, papers), duplicated data sources included from other reports (n=2, 3.3%, papers), and information about complementary and alternative medicines provided (n=12, 19.6%, papers). See Figure 1 for the PRISMA flowchart of the study selection process.

### Characteristics of Included Studies

In total, 26 studies were included in the systematic review [16,24-48] (Table 1). These studies were published between 2005 and 2021, with 20 (76.9%) studies in English [16,24-32,35,36,38-42,44,45,47] 3 (11.5%) in Chinese [33,46,48] 2 (7.7%) in Spanish [34,37], and 1 (3.8%) in Dutch [43]. Most of the studies were conducted in Asia (n=12, 46.2%) [27,30-33,38,39,41,45-48], followed by Europe (n=7, 26.9%) [16,28,29,35,36,42,43], North America (n=5, 19.2%) [24,25,34,37,44], and 1 (3.8%) each in Africa [40] and Australia [26]. In terms of the source of the reporter, AEs were reported only by HCPs in 4 (15.4%) studies [29,35,38,41], including physicians, pharmacists, nurses, or herbal practitioners; by HCPs, consumers, and other stakeholders (pharmaceutical companies, manufacturers) in 18 (69.2%) studies; and by only consumers in 2 (7.7%) studies [24,26]; the remaining 2 (7.7%) studies [34,37] had no information on reporters. Data of AEs were acquired from the spontaneous reporting system of the national PV center (n=21, 80.8%) [16,26,28-31,33,35-48], the regional PV center (n=3, 11.5%) [27,32,34], and the poison control center (n=2, 7.7%) [24,25].

**Table 1:** Characteristics of included studies (N=26).

Study	Country	Source of databases	Report collection duration	Source of the reporter
Mazzanti et al [16]	Italy	Phytovigilance system database from the Italian National Institute of Health	7 years	Anyone observing a suspected adverse reaction
Dennehy et al [24]	United States	California Poison Control System (CPCS)	6 months	Consumers
Gryzlak et al [25]	United States	American Association of Poison Control Center	1 year	HCPs^a^, consumers
Hoban et al [26]	Australia	Therapeutic Goods Administration (TGA)	14 years	HCPs
Huang et al [27]	China	Hubei Adverse Drug Reaction Monitoring Center	6 years	Medical institutions, enterprises, consumers
Ippoliti et al [28]	Italy	Italian Medicines Agency	18 years	Physicians, pharmacists, consumers
Jacobsson et al [29]	Sweden	Swedish Medical Products Agency (MPA)	19 years	Senior physicians (75%), pharmacists (4%), drug industry staff (2%), nurses (1%), dentists (0.3%)
Kalaiselvan et al [30]	India	National Coordination Centre (NCC) for the Pharmacovigilance Programme of India	2 years 6 months	HCPs, consumers, pharmaceutical companies
Lee et al [31]	Malaysia	Malaysian Adverse Drug Reactions Advisory Committee (MADRAC)	18 years	Pharmacists (n=196, 83.4%), physicians (n=27, 11.5%), pharmaceutical companies (n=2, 0.9%), others (n=10, 4.3%)
Li et al [32]	China	China Guangdong Provincial Center of ADR Monitoring, district hospitals	12 years	HCPs, manufacturers, consumers
Li et al [33]	China	National Drug ADR Monitoring Center	4 years	HCPs, consumers, others
Lores and Lazo [34]	Republic of Cuba	Provincial Coordinating Unit of Pharmacovigilance Santiago de Cuba	5 years	N/R^b^
Menniti-Ippolito et al [35]	Italy	Italian National Institute of Health	5 years	Hospital doctors (43%), pharmacists (22%), general practitioners (16%), specialist physicians (8%), herbalists (2%), others (5%), patients (1 report)
Petronijevic et al [36]	Republic of Serbia	National Pharmacovigilance Centre	14 years	Physicians (81.4%), pharmacists (5.7%), marketing authorization holders (12.9%)
Salvador et al [37]	Republic of Cuba	Cuban Pharmacovigilance System Vigibase	8 years	N/R
Saokaew et al [38]	Thailand	Health Products Vigilance Center (HPVC)	9 years	HCPs (100%)
Shin et al [39]	Republic of Korea	Korean Food and Drug Administration, Consumer Agency, Association of Traditional Korean Medicine	12 years	Consumers, pharmaceutical companies
Skalli et al [40]	Morocco	Moroccan Pharmacovigilance Herbal Center	9 years	N/R
Suwankesawong et al [41]	Thailand	Thailand Health Product Vigilance Center (HPVC)	1 year, 11 months	HCPs (100%)
Svedlund et al [42]	Sweden	Swedish Medical Products Agency	9 years	HCPs, patients
van Hunsel and van Grootheest [43]	Netherland	Dutch Updates Center Lareb	12 years	Pharmacies (44%), general practitioners (27%), consumers (14%), specialists (14%), hospital pharmacists (0.8%)
Wallace et al [44]	United States	Center for Food Safety and Applied Nutrition’s Adverse Event Reporting System (CAERS)	5 years	HCPs, consumers, manufacturers
Wechwithan et al [45]	Thailand	National pharmacovigilance database	12 years	HCPs, consumers
Wei et al [46]	China	National Adverse Reaction Monitoring Center	9 years	Reported by everyone
Xu et al [47]	Singapore	Vigilance & Compliance Branch of Health Sciences Authority	7 years	Doctors (87%), pharmacists (10%), traditional Chinese medicine practitioners (0.9%), manufacturers (1.1%), nurses (0.5%), others (0.3%)
Zhang et al [48]	China	National Center for Adverse Drug Reaction Monitoring	12 years	Reported by everyone

^a^HCP: health care professionals.

^b^N/R: not reported.

### Severity, Affected Body Systems, and Outcomes Involving AEs of HMs

Of the 26 studies reviewed, 24 (92.3%) provided information about the severity of AEs but only 9 (34.6%) studies specifically mentioned the assessment scale. Among these, the WHO scale was the most commonly used, mentioned in 4 (15.4%) studies [24,34,41,45], while 1 (3.8%) study used a modified Hartwig scale [26]. In contrast, 4 (15.4%) studies [25,38,42,43] defined their own severity classification, distinguishing between nonserious and serious events or categorizing them as minor, moderate, or major.

The most frequently affected systems as per the median reporting rate were (1) the skin and appendage (median 22.3%, IQR 13.3%-35.1%), with AEs such as rash, itching, erythema, urticaria, and pruritus [16,27-30,33-35,37,38,40-43,46,47]; (2) the gastrointestinal system (median 17.5%, IQR 9.0%-36.9%), with AEs such as abdominal pain, nausea, and vomiting [16,27-30,33-35,37,38,40-42,44,46,47]; (3) the central nervous system (median 12.5%, IQR 6.9%-17.9%), with AEs such as dizziness, fatigue, and headache [16,26-28,30,33-35,37,38,40-44,46,47]; and (4) the cardiovascular system (median 5.0%, IQR 1.8%-19.0%), with AEs such as hypertension, heart flutter, and tachycardia [16,28,29,35,37,40,41,44,47]. One study [36] reported AEs pertaining to the hepatobiliary system, including hepatitis (66.7%), hepatic necrosis (16.7%), and increasing hepatic enzymes (33.3%).

Of the 26 studies, 11 (42.3%) reported AE outcomes. The reported outcomes after treatment were “not recovered” (median 0.6%, IQR 0.3%-11.8%) in 5 (45.5%) studies [27,31,46-48], “recovered” (median 46.4%, IQR 12.5%-66.8%) in 10 (90.9%) studies [16,27,28,31-33,40,46-48], “cured” (median 54.2%, IQR 23.8%-63.8%) in 5 (45.5%) studies [27,32,33,46,48], and “fatal/death” (median 1.5%, IQR 0.7%-6.6%) in 6 (54.5%) studies [27,29,31,32,40,47]. Table S1 in Multimedia Appendix 1 shows a detailed summary.

### Causality Assessment Between AEs and HMs

Of the 26 studies, 16 (61.5%) reported the causality assessment scale used, where the WHO Uppsala Monitoring Centre (UMC) was the most common in 8 (50%) studies [16,29-31,34,36,40,42], followed by the Naranjo algorithm in 2 (12.5%) studies [38,43]; the Karch and Lasagna criteria in 1 (6.3%) study [37]; a combination of the WHO-UMC, the Naranjo algorithm, and the local Thai algorithm in 1 (6.3%) study [45]; and a predefined scale (definite, probable, possible, doubtful) in 1 (6.3%) study [24]. In addition, 3 (18.8%) of the 16 studies [33,46,48] conducted in China applied data mining methods, including the proportional reporting ratio (PRR) and the Bayesian confidence propagation neural network (BCPNN), for signal monitoring to detect the correlation between AEs and HMs (Table S1 in Multimedia Appendix 1).

The causality assessment ranged mostly from “possible” to “probable” (Table S1 in Multimedia Appendix 1). Notably, by using data mining methods (the PRR and the BCPNN), the 3 (18.8%) studies conducted in China could clarify the warning signals for specified HMs: *Dengzhan Xixin* (headache, dizziness, itching, chills, heart palpitations, fever, flushing) [33], *Guizhi Fuling* (gastric dysfunction, abdominal pain [46], and *Shujinjianyao* (rash, nausea, abdominal pain, headache, vomiting) [48].

### Quality of Information Provided in AE Reports

None of included studies provided full details of herbal products, with deficiency noted in the omission of batch numbers, identified as crucial elements [49]. Among the reviewed studies, a minority (n=11, 42.3%) mentioned specific brand names [16,24,27-30,33,35,43,46,48], 1 (3.8%) study identified the manufacturer [24], and 9 (34.6%) detailed the pharmaceutical form [27,28,32-34,40,41,46,48]. Furthermore, there was a notable absence of data pertaining to the quality assessment of herbal medicinal products, including information about testing for contamination, the presence of adulterants, or purity levels.

### Risk-of-Bias Assessment and Publication Bias

Applying Crombie’s checklist for ROB assessment, 3 (11.5%) of the 26 included studies [27,29,31] fulfilled most of the criteria and were classified as showing low ROB (6-7 points), 8 (30.8%) studies [26,28,32-34,37,45,47] showed a moderate ROB (>4 to <6 points), and 15 (57.5%) studies [16,24,25,30,35,36,38-44,46,48] were classified as showing a high ROB (≤4 points). For ROB assessments, the interrater agreement was high (Cohen κ=0.94).

**Figure 2:**
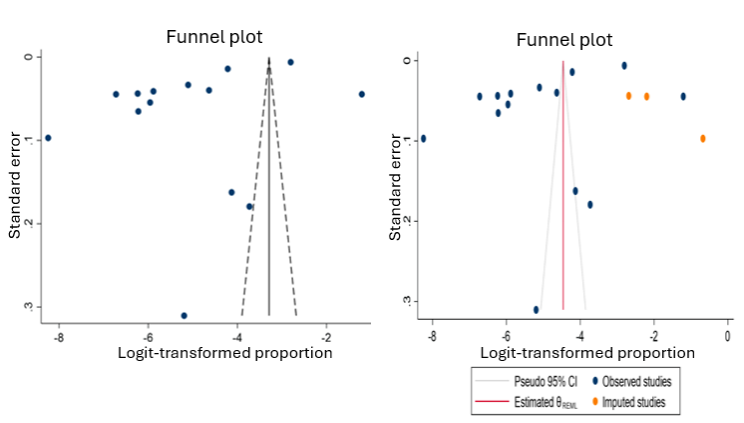
Funnel plots testing the publication bias before (left) and after (right) applying the trim-and-fill method.

The evidence of publication bias was detected by performing the Egger test (*P*=.01). The trim-and-fill method of calibrating the publication bias identified 3 (11.5%) imputed studies (Figure 2).

### Quantitative Synthesis

A total of 14 (53.8%) of the 26 studies providing sufficient data for calculating the reporting rate of AEs were submitted for meta-analyses.

#### Pooled Reporting Rate of AEs of HMs Using PV Databases

**Figure 3:**
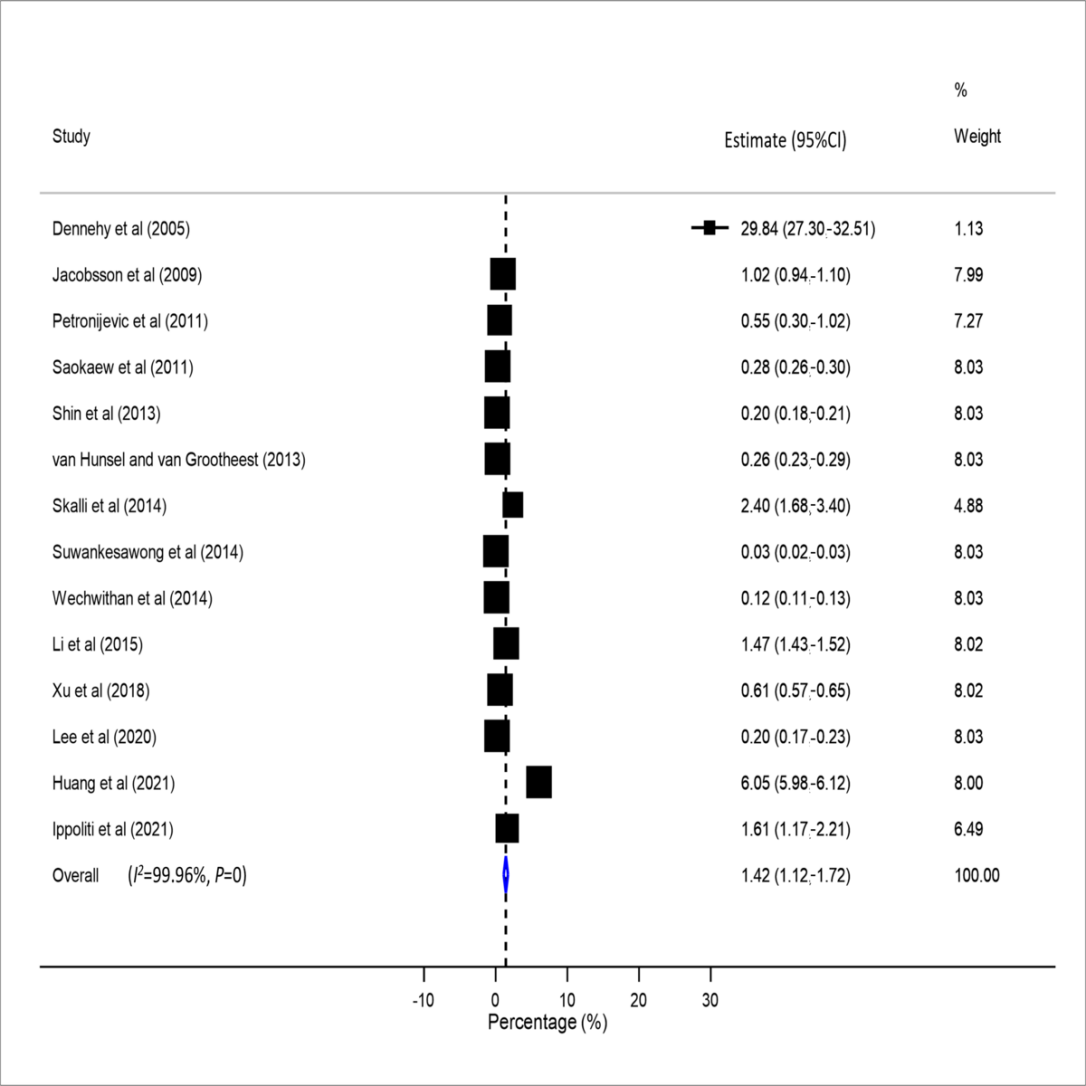
Forest plot showing the pooled reporting rate of the AEs associated with HMs. AE: adverse event; HM: herbal medicine.

The meta-analysis of the 14 (53.8%) studies showed a pooled reporting rate of the AEs of HMs at a median of 1.42% (IQR 1.12%-1.71%). There was significant heterogeneity (*χ*^2^_13_=33,650.09, *P*<.001, *I*^2^=99.96%) as the reporting rates ranged considerably from 0.03% to 29.84% (Figure 3).

#### Subgroup Analysis

A subgroup analysis by continent showed that there was a significant difference between Asia and Europe (Table 2), with median estimates of 1.12% (IQR 0.74%-1.50%) and 0.83% (IQR 0.28%-1.37%), respectively (*P*<.001). A subgroup analysis by ROB showed that “moderate ROB” studies detected a higher reporting rate compared to “high ROB” studies (Table 2), with median estimates of 0.95% (IQR 0.33%-1.57%) and 0.41% (IQR 0.25%-0.57%), respectively (*P*<.001).

**Table 2:** Subgroup analysis testing heterogeneity by continent, source of databases, source of the reporter, and ROB^a^.

Covariates		Studies (n=14), n (%)	Reporting rate estimate (%), median (IQR)	Heterogeneity test		
				*χ*^2^ (*df*)	*P* value	*I*^2^ (%)
**Continent**						
	Asia	8 (57.1)	1.12 (0.74-1.50)	32445.48 (7)	<.001	99.9
	Africa	1 (7.1)	2.40 (1.68-3.40)	—^b^	—	—
	Europe	4 (28.6)	0.83 (0.28-1.37)	350.85 (3)	<.001	99.1
	North America	1 (7.1)	29.84 (27.30-32.51)	—	—	—
**Source of databases**						
	Poison control center	1 (7.1)	29.84 (27.30-32.51)	—	—	—
	National PV^c^ center	11 (78.6)	0.40 (0.30-0.50)	2538.21 (10)	<.001	99.6
	Regional PV center	2 (14.3)	2.58 (2.54-2.62)	—	—	—
**Source of the reporter**						
	Consumer only	1 (7.1)	29.84 (27.30-32.51)	—	—	—
	HCPs^d^ only	3 (21.4)	0.44 (0.14-0.73)	1044.49 (2)	<.001	99.8
	All stakeholders	10 (71.4)	1.32 (0.80-1.85)	29250.89 (9)	<.001	99.9
**ROB**						
	High	7 (50.0)	0.41 (0.25-0.57)	1527.25 (6)	<.001	99.6
	Moderate	5 (35.7)	0.95 (0.33-1.57)	4475.91 (4)	<.001	99.9
	Low	2 (14.3)	0.84 (0.82-0.87)	—	—	—

^a^ROB: risk of bias.

^b^Not applicable.

^c^PV: pharmacovigilance.

^d^HCP: health care professional.

#### Meta-Regression Model

We conducted meta-regression analysis to identify potential sources of heterogeneity in the reporting rate of the AEs of HMs. The covariates considered included the continent, the source of the reporter, source of databases, and the ROB. From the model, we found that the source of the reporter was associated with the pooled reporting rate of the AEs of HMs (*P*=.01), whereas there was no relationship between the location, databases, or ROB and the reporting rate of AEs (Table 3).

**Table 3:** Results of univariate meta-regression analysis and the potential sources of heterogeneity in the reporting rate of AEs^a^ associated with HMs^b^.

Covariate	Coefficient (SE)	*t*_13_ (95% CI)	*P* value
Continent	0.032 (0.016)	1.94 (–0.004 to 0.668)	.076
Source of the reporter	–0.083 (0.026)	–3.19 (–0.140 to –0.026)	.008
Source of databases	–0.084 (0.040)	–2.08 (–0.173 to 0.004)	.059
ROB^c^	0.016 (0.029)	0.52 (–0.0492 to 0.803)	.061

^a^AE: adverse event.

^b^HM: herbal medicine.

^c^ROB: risk of bias.

#### Sensitivity Analysis

There was little difference in the pooled reporting rate when studies with a high ROB were added to the analysis. The reporting rate slightly decreased from 1.58% to 1.42%. In contrast, of the 14 (53.8%) studies included in the analysis, 1 (7.1%) [24] with only consumer reports impacted the pooled reporting rate. When this study [24] was added, the pooled reporting rate rose significantly from 1.09% to 1.42% (Table 4).

**Table 4:** Results of sensitivity analysis to examine the influence of studies with a high ROB^a^ and source of the reporter to the pooled estimate of the reporting rate of AEs^b^ associated with HMs^c^.

Analysis	Studies (n=14), n (%)	Reporting rate estimate (%), median (IQR)
All studies	14 (100.0)	1.42 (1.12, 1.72)
No studies with a high ROB	7 (50.0)	1.58 (0.68, 2.48)
No study with only consumer reports	13 (92.9)	1.09 (0.79, 1.39)

^a^ROB: risk of bias.

^b^AE: adverse event.

^c^HM: herbal medicine.

## Discussion

### Principal Findings

The increasing worldwide trend of HM usage reflects the growing number of people placing faith in the efficacy of HMs, emphasizing the urgent need to address concerns about the risks and benefits of HMs. This systematic review and meta-analysis critically assessed the reporting rate of AEs associated with HMs using data sourced from PV databases. Our findings underscore the presence of potential risks, alongside benefits, prompting continued concerns regarding the safety of HMs in clinical applications. The reported median rate of the AEs of HMs was calculated as 1.42%, with the highest AE reported rate of 29.84% and the reported outcomes of AEs being as serious as death (1.5%). With the increased trend in the consumption of HMs, an effective ongoing process to detect, assess, and prevent adverse effects from HMs is essential, allowing us to understand more about their benefits and risks. This understanding is crucial for promoting informed decision-making among health care providers and consumers, ensuring safer use of these products. Additionally, it facilitates the development of evidence-based guidelines and recommendations for integrating HMs into health care practices, while addressing gaps in knowledge regarding their pharmacological interactions and long-term effects.

The meta-regression and sensitivity analysis revealed a significant impact, with the highest reporting rate (median 29.84%, IQR 27.30%-32.51%) observed for AEs reports submitted by consumers. This highlights the importance of direct consumer reporting in identifying the adverse effects of HMs and underscores the necessity of integrating such reporting into the PV system [2]. Since the 20th century, numerous countries have established consumer reporting systems, with Canada pioneering this initiative in 1965 [50]. Various measures have been undertaken to motivate consumers to report, including providing feedback to reporters and promoting the reporting system through media, social media, and health care providers [50]. In addition, consumer organizations play a crucial role in bridging the gap between consumers and national PV centers, thereby fostering the use of these reporting systems [2,50]. However, since only medical professionals can accurately establish causality, relying solely on information provided by consumers does not validate the occurrence of an AE caused by a specific product [51]. Hence, effort is needed to develop and enhance more effective processes for collecting, detecting, and assessing the quality of consumer reports. First, the reporting form should be simplified for consumers to complete by using layperson language and including consumer-specific questions, especially other aspects of medicine use, such as experiences of ineffectiveness [52]. Their experiences might provide important insight, emphasizing aspects consumers are unable to communicate to their doctors [53]. Since there has been no standardized consumer reporting form for the AEs of HMs, the promotion and emphasis of international guidelines for a standard patient reporting form are essential [2,50]. Second, promotion and education of using the spontaneous reporting system for consumers are required, even though consumers may be aware of the self-reporting system and prepared to use it [2,54]. Third, a follow-up strategy should be implemented to obtain more medical confirmations, which can aid in analyzing the cause-and-effect relationship and ensuring the data elements of reports are as complete and accurate as possible [2].

A significant challenge in summarizing data in this systematic review and meta-analysis was the lack of comprehensive information about the HMs cited in the AE reports. Only the main ingredients were disclosed, while other potentially harmful components were not listed. Clearly stating all herbal components in AE reports is essential for ensuring accurate and comprehensive documentation of the potential risks associated with HMs [6]. Understanding specific botanical ingredients helps HCPs and researchers analyze adverse reactions more effectively. Identifying these components also aids in assessing interactions between herbs and medications, improving comprehension of AE mechanisms [15,55]. This knowledge supports creating clearer guidelines and warnings, enhancing transparency and reliability in reporting AEs, and promoting safer use and informed decision-making in the herbal product industry [6]. AE reports for HMs should include the brand name, manufacturer, pharmaceutical form, extract amount per dose, ingredients, excipients, regulatory status, and test results for contamination, adulterants, or purity [49].

Although numerous HMs and AEs were screened in this systematic review and meta-analysis, this is deemed insufficient, as many unregulated or self-prescribed HMs remain unaccounted for by existing PV systems [6]. Challenges in HM monitoring include diversity in the classification of HMs across nations, a lack of rigorous quality management, and insufficient collaboration among stakeholders. Therefore, a PV system for HMs should raise awareness of PV activities for HMs in the public domain. The involvement of all relevant stakeholders, including HCPs, consumers/patients, manufacturers, complementary practitioners, and sellers/distributors, is crucial for the effective engagement of the system in actively monitoring and reporting AEs [6,55].

### Strengths and Limitations

This study has many strengths, including a comprehensive literature search without language restrictions, double-data extraction, and ROB assessments. Our systematic review and meta-analysis also has some limitations. First, due to limited available publications, only 1 study targeting consumer reports was included in the quantitative analysis as a representative of the AE-reporting rate from consumer reports. The result therefore needs to be interpreted with caution. Additional studies gathering data on consumer reports should be conducted to obtain more accurate estimates of the reporting rate. Second, this systematic review and meta-analysis excluded studies on prevalence or incidence rates that did not collect AE reports from PV databases. Future studies should examine the prevalence rate of AEs associated with HMs in various settings (eg, hospitals, community pharmacies) to provide an overview of real-world data on these AEs.

### Conclusion

This systematic review and meta-analysis highlighted HM risks with a wide range of AE-reporting rates, depending on the source of the reporter, and revealed deficiencies in detailed HM component information provided within AE reports. Continuous efforts are necessary to standardize consumer reporting systems in terms of the reporting form, education, and follow-up strategy to improve data quality assurance measures, aiming to enhance the reliability and utility of PV data for monitoring the safety of HMs. Achieving effective monitoring and reporting of these AEs necessitates collaborative efforts from diverse stakeholders, including patients/consumers, manufacturers, physicians, complementary practitioners, sellers/distributors, and health authorities.

## Appendix

Multimedia Appendix 1 Summary of severity assessment, causality assessment, affected body systems, and outcomes of AEs of HMs. AE: adverse event; HM; herbal medicine.

Multimedia Appendix 2 PRISMA checklist for reporting systematic review.

## Abbreviations

AE: adverse event
BCPNN: Bayesian confidence propagation neural network
HCP: health care professional
HM: herbal medicine
PRISMA: Preferred Reporting Items for Systematic Reviews and Meta-Analyses
PRR: proportional reporting ratio
PV: pharmacovigilance
ROB: risk of bias
UMC: Uppsala Monitoring Centre
WHO: World Health Organization

## Data Availability

The data sets analyzed during this study are available from the corresponding author upon reasonable request.
