# Supplementary material for: Estimating Adverse Events Associated With Herbal Medicines Using Pharmacovigilance Databases: Systematic Review and Meta-Analysis
Source: JMIR Public Health Surveill. 2024 Aug 29;10:e63808. doi: 10.2196/63808 (PMC11393504; doi:10.2196/63808)
Supplement: Multimedia Appendix 1 [file publichealth_v10i1e63808_app1.docx]

**Table S1.** Summary of severity assessment, causality assessment, affected body systems, and outcomes of AEs^a^ of HMs^b^.

| Studies | Herbal components | Severity scales | Severity classification | Causality scale | Causality assessment | Affected body systems | AE outcomes |
| --- | --- | --- | --- | --- | --- | --- | --- |
| Mazzanti et al [1] | *Euterpe oleracea*, green tea d.e., anhydrous caffeine, *Carthamus tinctorius*, *Paullinia cupana*, *Centella asiatica L*, *Desmodium adscendens*, green tea leaves, fennel, ginger, dandelion root, red grapevine | N/R^c^ | Serious (64%), not serious (15%), no information (21%) | WHO-UMC^d^ | Certain (1.5%), probable (12%), possible (65%), unlikely (6%), and unclassifiable (15%) | Cardiovascular system (26%), liver (14%), gastrointestinal system (17%), central nervous system (12%), skin (9%) | Recovered (53%) |
| Dennehy et al [2] | *Ephedra* or non-*Ephedra* ingredients | WHO | No treatment required (15.5%), minor (13.9%), moderate (45.9%), severe (7.4%), lethal (0.3%) | Predefined scale (definite, probable, possible, doubtful) | Probable (49.2%), possible (30%) | N/R | N/R |
| Gryzlak et al [3] | *Echinacea* or St John’s wort (SJW) | Predefined approach (minor, moderate, major) | Echinacea: minor effect (2.8%), moderate effect (0%), potentially toxic (0.6%)  SJW: minor effect (8.5%), moderate effect (2.1%), potentially toxic (3.8%) | N/R | N/R | N/R | N/R |
| Hoban et al [4] | SJW *(Hypericum perforatum)* | Modified Hartwig scale | Mild (38.1%), moderate (44.1%), severe (11.9%), life-threatening (6%) | N/R | N/R | Majority of cases affecting the central nervous system (45.2%) | N/R |
| Huang et al [5] | *Shenmai* injection, *Xiangdan* injection, *Qingkailing* injection, Panax ginseng saponins injection, Salvia injection, *Tanreqing* injection safflower injection, *Xueshuantong* injection, *Xiyanping* injection, *Shengmai* injection, Astragalus injection, *Reduning* injection, *Dan hong* injection, *Shuanghuanglian* injection, *Xuebijing* injection | N/R | Serious (12.6%), nonserious (87.4%) | N/R | N/R | Skin and appendage (36.4%), the body as a whole (24.5%), nervous system (12.5%), gastrointestinal system (9.6%) | Relieved (45.1%), cured (54.2%), cot relieved (0.3%), death (0.04%) |
| Ippoliti et al [6] | Herbal substances claiming tonic or adaptogenic properties, SJW, herbal products containing caffeine (green tea and guarana) | N/R | Serious (40%), including hospitalization (36.2%), life threatening (1.7%), and disability (1.7%); not serious (60%) | N/R | N/R | Skin (21%), cardiovascular system (21%), digestive system (17%), liver (17%), central nervous system (14%) | Recovered (69%), persistent reaction (12.1%), improved (1.8%) |
| Jacobsson et al [7] | Purple coneflower (*Echinacea purpurea*; 8.1%), purple coneflower + Siberian ginseng (*Eleutherococcus senticosus*) + Malabar nut (*Adhatoda vasica*; 7.3%), ginkgo leaf (*Ginkgo biloba*; 6.7%), SJW (*H. perforatum*; 5.6%) | N/R | Serious (28.4%), fatal (1.41%) | WHO-UMC | Possible (4.5%) | Skin (31.2%), liver and biliary system (17.9%), blood (5.1%), cardiovascular system (3.2%), gastrointestinal system (3%) | Fatal (1.4%) |
| Kalaiselvan et al [8] | Senna extract, aloe, mustard oil, digitalis, garlic, menthol, and turmeric | N/R | Serious (48.7%), nonserious (30.8%), unknown (28.2%) | WHO-UMC | Certain (15.9%), probable 46.2%), possible (25%), unlikely (9.8%), conditional (0.8%) | Skin and appendages (52.5%), gastrointestinal system (35%), central and peripheral nervous systems (7.5%), respiratory system (7.5%) | N/R |
| Lee et al [9] | Black seed “habbatus sauda” (*Nigella sativa*), pomegranate (*Punica granatum*), ginger (*Zingiber officinale*) | N/R | Serious (47.2%), including death (3.8%), life threatening (0.9%), causing or prolonging hospitalization (42.6%); not serious (5.1%); unspecified (47.7%) | WHO-UMC | Possible (100%) | Hepatic disorder (100%) | Recovered (10.6%), recovering (17.0%), not recovered (10.2%), fatal/death (5.5%), unspecified (56.6%) |
| Li et al [10] | *Gingkailing* injection (QI): mixture of *Concha margaritifera*, *Fructus gardenia*, *Cornu bubali*, *Radix isatidis*, *Flos lonicerae*, cholalic acid, hyodeoxycholic acid, baicalin | N/R | Serious (13.1%), moderate (87%) | N/R | N/R | N/R | Cured (72.2%), getting better (27.1%), recovering with sequelae (0%), death (0.7%) |
| Li et al [11] | *Dengzhan Xixin* | N/R | N/R | PRR^e^, BCPNN^f^ | N/R | Skin (25.1%), systemic damage (18.6%), nervous system (16.2%), gastrointestinal system (8.9%) | Cured (55.3%), improved (39%) |
| Lores and Lazo [12] | HM products (unspecified herbal components) | WHO | Mild (74.2%), moderate (25.8%) | WHO-UMC | Probable (49.2%), possible (37%) | Digestive system (26.4%), skin (23.4%), cardiovascular system (21.1%), central nervous system (18.5%) | N/R |
| Menniti-Ippolito et al [13] | *Pinus* sp., *Eriodictyon californicum*, *Chelidonium majus*, *Coleus forskolii*, *Citrus aurantium*, *magnolia*, *C. aurantium*, magnolia, *Araxacum officinale*, *Morinda citrifolia*, *Rhodiola rosea*, *Cassia angustifolia*, *Achillea millefolium*, *Aloe vera*, *Lavandula vera*, *C. reticulata* | N/R | Hospitalization (35%), life threatening (6%), fatal (2 events) | N/R | N/R | Skin (19%), gastrointestinal system (18%), psychiatric disorders (10%), nervous system (8%) | N/R |
| Petronijevic et al [14] | Chinese multicomponent herbal products (*Angelicae Dahuricae radix*, *Notopterygii rhyzoma seu radix*, *Gastrodiae* rhizome, *Chuanxiong* rhizome, *Bambix morii*, *Corydalis* rhizome, *Achyranthis radix*, *Puerarie radix*, *Bupleuri radix*) | N/R | Hospitalized (60%) | WHO-UMC | N/R | Hepatic system (100%) | N/R |
| Salvador et al [15] | *Allium sativum L.* (garlic tincture), *A. vera L.* (Sabila syrup), *Cymbopogon citratus* (Canasanta tincture), *Eucalyptus* spp. (eucalyptus syrup and tincture), *Plecthrantus* amboinicus (Lour.) *Spreng* (oregano syrup), *C. sinensis* [L.] *Osbeck* (sweet orange dye), *Peppermint L*. (peppermint), *Z. officinale* R (ginger tincture), *Calendula officinalis* L. (calendula tincture), *Pedilanthus* *tithymaloides* (L.) *Poit* (Itamo real) | N/R | Mild (65.4%), moderate (34.5%), serious (0.1%), lethal (0%) | Karch and Lasagna criteria | Definitive (1.6%), probable (64.7%), possible (20.5%), conditional (13.1%), not related (0.1%) | Gastrointestinal system (47.8%), skin (18.1%), cardiovascular system (11.9%), central nervous system (5.2%) | N/R |
| Saokaew et al [16] | Thai HMs (unspecified herbal components) | Predefined approach (nonserious or serious) | Serious (4.2%), nonserious (90.4%) | Naranjo | Probable (0.2%), possible (99.8%) | Gastrointestinal system (37.8%), body as a whole (general disorders; 14.4%), psychiatric disorders (12.5%), central and peripheral nervous systems (12.3%), skin and appendages (10.5%) | N/R |
| Shin et al [17] | *Ephedra sinica*, *Erigeron canadensis*, *Pinellia ternata*, *Xanthium strumarium*, *Evodia rutaecarpa*, *Prunus armeniaca*, *P. persica*, *Sinomenium acutum* | N/R | N/R | N/R | N/R | Hepatitis (46.8%) | N/R |
| Skalli et al [18] | *Juniperus oxycedrus* | N/R | Hospitalization (100%) | WHO-UMC | Probable (13%), possible (80%), unlikely (3.3%) | Central and peripheral nervous systems (25.5%), respiratory system (19.6%), psychiatric disorders (11.8%), urinary system (9.8%), blood (7.8%), skin and appendages (5.9%) | Improved and discharged (76.7%), unknown (13.3%), deaths (10%) |
| Suwankesawong et al [19] | *Andrographis paniculata* | WHO | Serious (17%), nonserious (83%) | N/R | Certain (7.5%), probable (57.6%), possible (31.1%), unlikely (0), unknown (3.8%) | Skin and appendages (51.9%), body as a whole (general; 23.5%), gastrointestinal system (9.1%), respiratory system (6.2%) | N/R |
| Svedlund et al [20] | Black cohosh rhizome (*Cimicifuga racemosa L. Nutt.*), purple coneflower herb (*E. purpurea L. Moench*), pollen of maize, rye, cock’s-foot, pine (*Zea mays L.*, *Secale cereal L.*, *Dactylis glomerata L.*, *Pinus sylvestris L.a*), gentian root (*Gentiana lutea L.*), *Primula* spp. flowers, sorrel herb (*Rumex* spp.), elderflower (*Sambucus nigra L.*), verbena herb (*Verbena officinalis L*.) | Predefined approach (nonserious or serious) | Nonserious (70.7%), serious (29.3%) | WHO-UMC | Probable (2.6%), possible (97.4%) | Skin and subcutaneous tissue (25.6%), gastrointestinal system (23.2%), nervous system (16%) | N/R |
| van Hunsel and van Grootheest [21] | *Plantago ovata* (psyllium), *H. perforatum* (SJW), *G. biloba* (ginkgo), *Valeriana officinalis* (*valeriaan*), *Thymus vulgaris* (*tijm*), *Actaea racemosa* (*zilverkaars*), *Senna alexandrina* (*senna*), *Glycyrrhiza glabra* (zoethout), *Althaea officinalis* (*heemst*), *E. purpurea* (rode *zonnehoed*), *Camellia sinensis* (*groene* *thee*), *Pimpinella anisum* (*anijs*), *Avena sativa* (haver), *E. sinica* (*ma huang*), *Equisetum* sp. (*paardenstaart*), *Ledum palustre* (moerasrozemarijn), *Symphytum officinale* (*smeerwortel*), *Rhus toxicodendron* (gifsumak), *Viscum album* (*maretak)*, *Cardiospermum halicacabum* (*blaasjeswingerd*), *Panax ginseng* (ginseng*)*, *Humulus lupulus* (hop*)*, *Melaleuca alternifolia* (*theeboom*, “tea tree”) | Predefined approach (nonserious or serious) | Serious (16.4%) | Naranjo | N/R | Skin and subcutaneous and gastrointestinal system (21.2%), central nervous system (17.3%), psychiatric disorders (13.7%), body in general (13.4%) | N/R |
| Wallace et al [22] | *Echinacea*, ginseng, garlic, *G. biloba*, SJW, peppermint | N/R | N/R | N/R | N/R | Gastrointestinal system (46.5%), neurologic (45.1%), miscellaneous (40.9%), blood/hepatic (24%), cardiovascular system (16.9%) | N/R |
| Wechwithan et al [23] | Thai traditional medicines (unspecified herbal components) | WHO | Serious (67.7%) | WHO-UMC, Naranjo, or a local Thai algorithm | N/R | N/R | N/R |
| Wei et al [24] | *Guizhi*, *Fuling*, *Mudanpi*, *Taoren*, *Baishao* | N/R | Severe (0.7%) | PRR, BCPNN | N/R | Gastrointestinal system damage (69.8%), skin and its accessories (10.3%), systemic damage (6.5%), central and peripheral system damage (4.4%) | Cured (48.9%), improved (47.6%), not improved (0.3%), not recorded (3.1%) |
| Xu et al [25] | Chinese HMs (unspecified herbal components) | N/R | Serious (50%) with 14 deaths, hospitalized (17.2%), not hospitalized (10.2%), no information (70.9%) | N/R | N/R | Skin and appendages (45%), body as a whole (general; 11.7%), metabolic and nutritional disorders (7.0%), liver and biliary system (6.3%) | Recovered (12.5%), not recovered (13.4%), uncertain outcome (68.0%), death (1.6%) |
| Zhang et al [26] | Dog ridge, golden cherry seed, chicken blood vine, *Qianjinba*, black tiger, *Niu Dali*, *Ligustrum lucidum* (steamed), mulberry parasitic (steamed), dodder (salt), Corydalis (made) | N/R | Severe (15.7%) | BCPNN | N/R | Skin and its accessories, central and peripheral nervous systems, hepatobiliary system | Cured (30.1%), improved (65.7%), not improved (0.6%), had sequelae (0.6%), unknown information (3.1%) |

^a^AE: adverse event.

^b^HM: herbal medicine

^c^N/R: not reported.

^d^WHO-UMC: World Health Organization Uppsala Monitoring Centre.

^e^PRR: proportional reporting ratio.

^f^BCPNN: Bayesian confidence propagation neural network.

## References

1. Mazzanti G, Vitalone A, Da Cas R, Menniti-Ippolito F. Suspected adverse reactions associated with herbal products used for weight loss: spontaneous reports from the Italian phytovigilance system. *Eur J Clin Pharmacol* 2019 Nov 19; 75(11):1599-1615.
2. Dennehy CE, Tsourounis C, Horn AJ. Dietary supplement-related adverse events reported to the California Poison Control System. *Am J Health Syst Pharm* 2005 Jul 15; 62(14):1476-1482.
3. Gryzlak BM, Wallace RB, Zimmerman MB, Nisly NL. National surveillance of herbal dietary supplement exposures: the poison control center experience. *Pharmacoepidemiol Drug Saf* 2007 Sept 06; 16(9):947-957.
4. Hoban CL, Byard RW, Musgrave IF. A comparison of patterns of spontaneous adverse drug reaction reporting with St. John's Wort and fluoxetine during the period 2000-2013. *Clin Exp Pharmacol Physiol* 2015 Jul; 42(7):747-751.
5. Huang R, Cai Y, Yang L, Shangguan X, Ghose B, Tang S. Safety of traditional Chinese medicine injection based on spontaneous reporting system from 2014 to 2019 in Hubei Province, China. *Sci Rep* 2021 Apr 23; 11(1):8875.
6. Ippoliti I, Menniti-Ippolito F, Mazzanti G, Di Giacomo S. Suspected adverse reactions to performance enhancing dietary supplements: spontaneous reports from the Italian phytovigilance system. *Phytother Res* 2021 Jun 10; 35(6):3246-3261.
7. Jacobsson I, Jönsson AK, Gerdén B, Hägg S. Spontaneously reported adverse reactions in association with complementary and alternative medicine substances in Sweden. *Pharmacoepidemiol Drug Saf* 2009 Nov 03; 18(11):1039-1047.
8. Kalaiselvan V, Saurabh A, Kumar R, Singh G. Spontaneous reporting of adverse events due to herbal products in Pharmacovigilance Programme of India. 2014. Presented at: 14th ISoP Annual Meeting on New Ideas in Ancient Cultures - Advancing; October 19-22, 2014; Tianjin, China.
9. Lee FY, Wong H, Chan H, Mohamed Ali N, Abu Hassan MR, Omar H, Abdul Mutalib NA. Hepatic adverse drug reactions in Malaysia: an 18-year review of the national centralized reporting system. *Pharmacoepidemiol Drug Saf* 2020 Dec 02; 29(12):1669-1679.
10. Li H, Deng J, Yue Z, Zhang Y, Sun H. Detecting drug-herbal interaction using a spontaneous reporting system database: an example with benzylpenicillin and qingkailing injection. *Eur J Clin Pharmacol* 2015 Sept 11; 71(9):1139-1145.
11. Li Y-Y, Xie Y-M, Shen H, Xiang Y-Y. Study of analysis 1 390 adverse drug reactions cases of parenterally administered Dengzhan Xixin based on China’s spontaneous response system. *CJCMM* 2013 Sept 15; 18(38):2998-3002.
12. Lores D D, Lazo R Y. Mexican Journal of Pharmaceutical Sciences. 2011 Mar. [Characterization of suspected adverse reactions to herbal medicines reported to the Provincial Coordinating Unit of Pharmacovigilance Santiago de Cuba]. https://www.scielo.org.mx/scielo.php?script=sci_arttext&pid=S1870-01952011000100004 [accessed 2024-08-20]
13. Menniti-Ippolito F, Mazzanti G, Santuccio C, Moro PA, Calapai G, Firenzuoli F, Valeri A, Raschetti R. Surveillance of suspected adverse reactions to natural health products in Italy. *Pharmacoepidemiol Drug Saf* 2008 Jun 10; 17(6):626-635.
14. Petronijevic M, Ilic K, Suzuki A. Drug induced hepatotoxicity: data from the Serbian pharmacovigilance database. *Pharmacoepidemiol Drug Saf* 2011 Apr 02; 20(4):416-423.
15. Salvador AKR, Milian A, Carbonell LA, López GJ, Orta IA, Lee AC. Vigilance of adverse reactions of herbal medicines in Cuba for the period 2003-2010. *Rev Cubana Plant Med* 2015; 20(1):14-24.
16. Saokaew S, Suwankesawong W, Permsuwan U, Chaiyakunapruk N. Safety of herbal products in Thailand: an analysis of reports in the Thai Health Product Vigilance Center database from 2000 to 2008. *Drug Saf* 2011 Apr 01; 34(4):339-350.
17. Shin H, Jeong S, Lee MS, Ernst E. Adverse events attributed to traditional Korean medical practices: 1999–2010. *Bull World Health Org* 2013 May 31; 91(8):569-575.
18. Skalli S, Chebat A, Badrane N, Bencheikh RS. Side effects of cade oil in Morocco: an analysis of reports in the Moroccan herbal products database from 2004 to 2012. *Food Chem Toxicol* 2014 Feb; 64:81-85.
19. Suwankesawong W, Saokaew S, Permsuwan U, Chaiyakunapruk N. Characterization of hypersensitivity reactions reported among Andrographis paniculata users in Thailand using Health Product Vigilance Center (HPVC) database. *BMC Complement Altern Med* 2014 Dec 24; 14(1):515.
20. Svedlund E, Larsson M, Hägerkvist R. Spontaneously reported adverse reactions for herbal medicinal products and natural remedies in Sweden 2007-15: report from the Medical Products Agency. *Drugs Real World Outcomes* 2017 Jun 28; 4(2):119-125.
21. van Hunsel FPAM, van Grootheest ACK. [Adverse reactions to herbal remedies: analysis of reported adverse reactions in the Netherlands]. *Ned Tijdschr Geneeskd* 2013; 157(47):A6615.
22. Wallace RB, Gryzlak BM, Zimmerman MB, Nisly NL. Application of FDA adverse event report data to the surveillance of dietary botanical supplements. *Ann Pharmacother* 2008 Apr 15; 42(5):653-660.
23. Wechwithan S, Suwankesawong W, Sornsrivichai V, McNeil EB, Jiraphongsa C, Chongsuvivatwong V. Signal detection for Thai traditional medicine: examination of national pharmacovigilance data using reporting odds ratio and reported population attributable risk. *Regul Toxicol Pharmacol* 2014 Oct; 70(1):407-412.
24. Wei R-L, Xie Y-M, Zhang W-L. [Analysis on 1 500 adverse reactions of Guizhi Fuling Capsules based on spontaneous response system]. *Zhongguo Zhong Yao Za Zhi* 2019 Apr; 44(7):1497-1502.
25. Xu Y, Patel DN, Ng SP, Tan S, Toh D, Poh J, Lim AT, Chan C, Low M, Koh H. Retrospective study of reported adverse events due to complementary health products in Singapore from 2010 to 2016. *Front Med (Lausanne)* 2018 Jun 12; 5:167.
26. Zhang C, Li P-P, Wang L-X, Xie Y-M, Li L. [ADR/AE early warning analysis of Shujin Jianyao Pills based on spontaneous reporting system]. *Zhongguo Zhong Yao Za Zhi* 2020 Aug; 45(15):3533-3538.
